# Composition and Legal Aspects of Reptiles and Amphibians Displayed at an Exotic Pet Fair in Warsaw (Poland)

**DOI:** 10.3390/ani16081138

**Published:** 2026-04-09

**Authors:** Damian Zieliński, Piotr Nawłatyna, Zofia Wójcik

**Affiliations:** Department of Animal Ethology and Wildlife Management, University of Life Sciences in Lublin, Akademicka 13, 20-950 Lublin, Poland; piotr.nawlatyna@up.edu.pl (P.N.); zwojcik_4a@wp.pl (Z.W.)

**Keywords:** exotic pet trade, captive breeding, reptiles, amphibians, species diversity

## Abstract

The global exotic pet trade has expanded in recent decades, raising concerns related to animal welfare, biodiversity conservation, and compliance with international regulations. Reptiles and amphibians constitute a major component of this trade, yet information on species availability and trade practices at exotic pet fairs remains limited. The primary aim of this study was to identify the reptile and amphibian species offered for sale at an exotic pet fair in Warsaw, Poland. Secondary objectives were to assess the declared origin of the animals and the availability of information regarding their legal and conservation status. Photographic documentation of all exhibition tables was used to record species identity, number of individuals, and labeling practices. In total, 818 individuals representing 74 species from 31 families were recorded. Reptiles, particularly squamates, dominated the assemblage, while amphibians accounted for a smaller proportion of the animals offered. More than half of the individuals were labeled as captive-bred, whereas only a small fraction were identified as imported or wild-caught; however, information on origin was absent for nearly half of the animals. Over 50% of the recorded species were listed in Appendix II of the Convention on International Trade in Endangered Species of Wild Fauna and Flora, yet no visible information on legal or conservation status was provided at the point of sale. These findings indicate that inconsistent labeling limits transparency and informed decision-making by buyers.

## Simple Summary

The trade in exotic reptiles and amphibians has expanded in recent years, but information provided to buyers at the point of sale is often limited. This study aimed primarily to identify which reptile and amphibian species are available in the exotic pet trade and, secondly, to assess the declared origin and legal status of the animals offered for sale. The research was carried out at an exotic pet fair in Warsaw, Poland. All sales tables were documented using photographs, allowing the number of animals, species identity, and information displayed by sellers to be recorded. More than 800 animals representing over 70 species were observed. Reptiles were offered more frequently than amphibians, with lizards and snakes being the most common groups. Although more than half of the animals were described as bred in captivity, nearly half had no information about their origin. Many of the species recorded are subject to international trade regulations, yet this was not communicated clearly to buyers. Limited and inconsistent labeling reduces transparency and may affect animal care after purchase. Providing clear and standardized information at the point of sale could improve animal welfare, support responsible ownership, and strengthen compliance with existing regulations.

## 1. Introduction

The global trade in exotic pets has grown over recent decades, raising concerns regarding animal welfare, biodiversity conservation, and compliance with international regulations [1,2,3,4,5,6,7]. Reptiles and amphibians are among the most frequently traded taxa due to their diversity and increasing popularity among hobbyists [2]. However, the demand for exotic species can lead to overexploitation of wild populations, illegal trade, and the spread of invasive species and pathogens in native ecosystems [2,4].

To reduce these risks, international agreements such as the Convention on International Trade in Endangered Species of Wild Fauna and Flora (CITES) regulate the trade of selected species, while captive breeding has been promoted as a more sustainable alternative to wild collection. In Europe, exotic pet fairs represent an important component of the legal trade, providing a platform for breeders to sell animals directly to consumers [8]. Despite their popularity, such events remain poorly studied, particularly with regard to species composition, origin of animals, and transparency of information provided to buyers [9,10].

To date, no studies in Poland have investigated which species are present in the Polish exotic pet trade, including both exotic pet fairs and other trade channels. Related research has focused exclusively on invasive alien species and potentially invasive species, specifically crayfish and turtles [11,12], as well as the online trade in non-native amphibians [13]. In other European countries, the diversity of species kept in amateur breeding and private collections has also been relatively poorly studied. Only a limited number of studies from neighboring countries are available, which could provide some indication of what might be expected in Poland [4,10]. However, none of these studies address animals displayed specifically at exotic pet fairs. Basic information can be found in the 2023 report by Eurogroup for Animals [8], although it primarily concerns the availability of entire taxonomic classes and invertebrates in general at various fairs. From an animal science perspective, the quality of information provided at the point of sale directly influences husbandry standards, survival, and long-term welfare of reptiles and amphibians kept in captivity [9,14,15,16].

The aim of this study was therefore to characterize the diversity, taxonomic composition, origin, and conservation status of reptiles and amphibians exhibited at an exotic pet fair in Warsaw (hereafter referred to as an exotic pet exposition). By documenting the species offered for sale and evaluating labeling practices, this study provides insight into the current state of the exotic animal trade in Poland and identifies potential gaps in regulation and transparency.

## 2. Materials and Methods

The study was performed at one of the exotic pet fairs which was held in Warsaw in 2023. The name of the event organizer is not disclosed due to the sensitive nature of the exotic pet trade sector in Poland, including ongoing regulatory discussions and competition between organizers. The event was a large, publicly accessible (ticketed event) exotic pet fair held in Warsaw in 2023 and is considered representative of this type of event in Poland. At this event, reptile and amphibian breeders were able to show their animals to the public. On the exhibit tables, animals were kept individually in exhibition boxes, with some information about the species’ name and other basic details (age, sex, source, whether they are captive bred or wild caught). Before the event was opened to the public, photographs of each exhibit table (including each box containing an individual animal) were taken for later evaluation. All photographs were taken by the same three observers using a standardized protocol to ensure consistency of data collection. The same observers were responsible for subsequent species identification and for verifying the accuracy of species names provided by exhibitors, including cases where discrepancies between labeling and actual species identity were detected. Species identification was based on visible morphological characteristics and the information provided on the labels. Scientific names and taxonomic classification of species were verified using the Encyclopedia of Life database. Information on the conservation and trade status of species was obtained from the current version of the Appendices of the Convention on International Trade in Endangered Species of Wild Fauna and Flora (CITES), available on the official CITES website (https://www.cites.org; based on the version of the CITES Appendices valid in 2023; accessed on 15 January 2026). It should be noted that only visibly presented animals were evaluated, so the potentially hidden ones were not taken into consideration. The study was non-invasive and based solely on visual documentation; no handling or disturbance of animals occurred. The results are shown as number of specimens (N) and percentage values (%) of each taxonomic group.

## 3. Results

### 3.1. Characterization of the Animals Available at the Fair

The species affiliation of the animals displayed on tables during the fair was determined. A total of 818 animals were displayed, of which 688 were reptiles (84.11%) and 130 were amphibians (15.89%). They represented 74 species from 31 families, distributed across 4 orders. The exact characteristics and systematic affiliation are shown in Supplementary Table S1.

**Figure 1:**
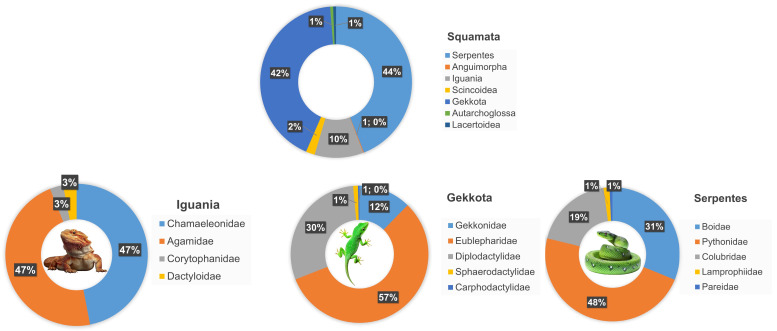
Systematic characteristics of animals displayed for sale during the fair (%), expressed as the percentage share of families.

Among the animals presented, the vast majority of individuals belonged to the order Squamata (78.37%); the others were Anura (13.72%), Testudinata (7.66%) and Caudata (0.25%). The most represented group of reptiles, i.e., Squamata, is shown in Figure 1. Among the Squamata, animals from the following suborders were exhibited for purchase at the fair: Anguimorpha (1; 0.16%; *Varanus exanthematicus*), Autarchoglossa (4; 0.63%; *Takydromus dorsalis*), Gekkota (268; 42.27%), Iguania (66; 10.41%), Lacertoidea (4; 0.63%; *Lacerta erhardii*, *Podarcis tauricus*), Scincoidea (12; 1.89%; *Mochlus fernandi*, *Gerrhosaurus nigrolineatus*), and Serpentes (279; 44.01%).

The Gekkota were represented by Eublepharidae (152; 56.72%; *Eublepharis macularius*), Diplodactylidae (79; 29.48%; *Correlophus auriculatus*, *Correlophus sarasinorum*, *Eurydactylodes vieillardi*, *Correlophus ciliatus*), Gekkonidae (33; 12.31%; *Phelsuma grandis*, *Phelsuma klemmeri*, *Phelsuma laticauda*, *Phelsuma lineata*, *Uroplatus henkeli*, *Lepidodactylus lugubris*), Sphaerodactylidae (3; 1.12%; *Gonatodes albogularis*) and Carphodactylidae (1; 0.37%; *Nephrurus levis*).

The Iguania were represented by Agamidae (31; 46.97%; *Pogona vitticeps*, *Pogona henrylawsoni*, *Physignathus cocincinus*), Chamaeleonidae (31; 46.97%; *Chamaeleo calyptratus*, *Trioceros jacksonii*, *Furcifer pardalis*, *Kinyongia boehmei*), Corytophanidae (2; 3.03%; *Basiliscus plumifrons*, *Corytophanes hernandezii*), and Dactyloidae (2; 3.03%; *Chamaeleolis porcus*, *Anolis biporcatus*).

The Serpentes were represented by families: Boidae (87; 31.18%; *Boa imperator*, *Corallus hortulanus*, *Eryx colubrinus*, *Epicrates cenchria* and hybrid *Epricrates cenchria* × *Epicrates maurus*), Colubridae (53; 19.00%; *Pantherophis guttatus*, *Heterodon nasicus*, *Lampropeltis californiae*, *Lampropeltis triangulum nelsoni*, *Lampropeltis triangulum campbelli*, *Ahaetulla prasina*, *Lycodon capucinus*, *Xenochrophis vittatus*), Lamprophiidae (4; 1.43%; *Boaedon fuliginosus*), Pareidae (2; 0.72%; *Pareas carinotus*), and Pythonidae (133; 47.67%; *Python regius*, *Malayopython reticulatus*, *Python bivittatus*, *Morelia spilota*, *Morelia spilota cheynei*).

**Figure 2:**
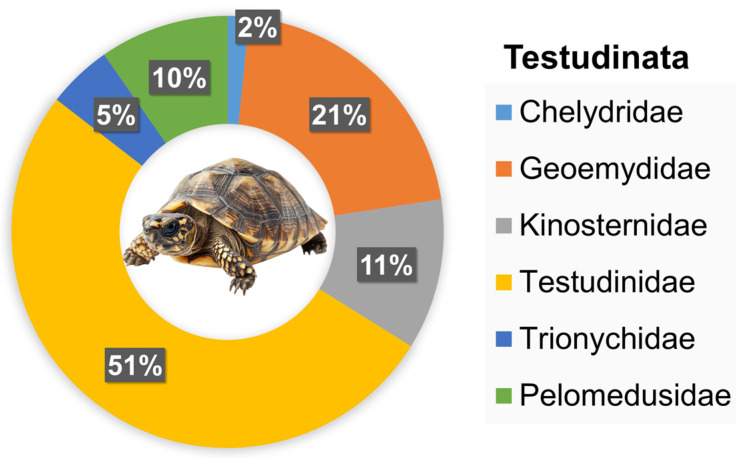
Taxonomic composition of Testudinata species recorded in studied exotic pet trade in Warsaw (Poland), expressed as the percentage share of families.

Testudinata was represented by two suborders (Figure 2), Cryptodira (56; 90.32%) and Pleurodira (6; 9.68%). The Cryptodira suborder was represented by Chelydridae (1; 1.79%; *Macrochelys temminckii*), Geoemydidae (13; 23.21%; *Mauremys sinensis*), Kinosternidae (7; 12.50%; *Sternotherus odoratus*), Testudinidae (32; 57.14%; *Centrochelys sulcata*, *Testudo hermanni*, *Testudo horsfieldii*, *Stigmochelys pardalis*), and Trionychidae (3; 5.36%; *Pelodiscus sinensis*). The Pleurodira suborder was represented by 2 species of the family Pelomedusidae: *Pelomedusa subrufa* and *Pelusios castaneus*.

**Figure 3:**
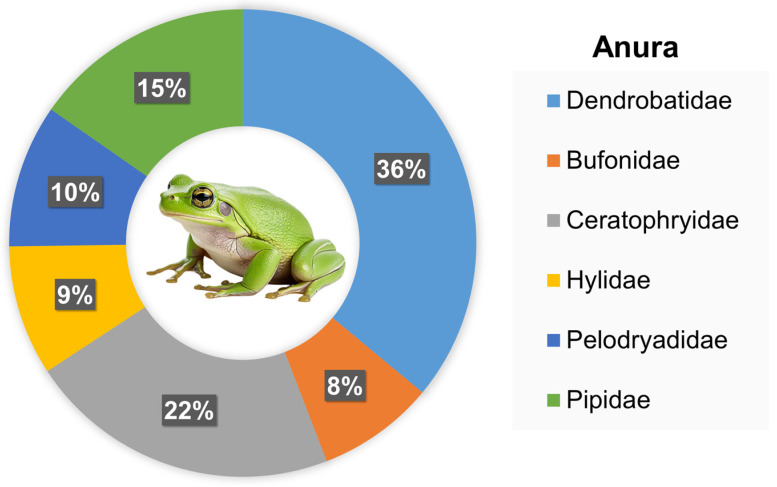
Taxonomic composition of Anura species recorded in studied exotic pet trade in Warsaw (Poland), expressed as the percentage share of families.

Among amphibians, Anura was represented by Mesobatrachia (17; 15.32%; *Hymenochirus boettgeri*) and Neobatrachia (94; 84.68%) (Figure 3), while Caudata was represented only by the suborder Salamandroidea (2 individuals of *Ambystoma tigrinum*). The Neobatrachia suborder was represented by Bufonidae (9; 9.57%; *Sclerophrys mauritanica*, *Rhinella marina*, *Incilius luetkenii*, *Duttaphrynus melanostictus*), Ceratophryidae (24; 25.53%; *Ceratophrys cranwelli*), Dendrobatidae (40; 42.55%; *Ranitomeya ventrimaculata*, *Adelphobates galactonotus*, *Dendrobates bicolor, Dendrobates tinctorius*, *Dendrobates auratus*,), Hylidae (10; 10.64%; *Trachycephalus resinifictrix*), and Pelodryadidae (11; 11.70%; *Litoria caerulea*).

Species recorded at the studied exotic pet fair originated from a wide range of geographic regions across multiple continents. The assemblage was dominated by taxa from Central and South America, Africa (including Madagascar) and Asia, which together comprised 68.9% of all recorded species. A smaller proportion of species originated from Australia and Oceania (14.9%). In contrast, species from temperate regions were less represented, with North American taxa accounting for 12.2% and European species constituting only 4% of the total. Most reptiles and amphibians represented taxa naturally occurring in tropical regions, especially in Central and South America, sub-Saharan Africa and Southeast Asia (approximately 80–90%, depending on classification). The predominance of tropical species, with European taxa accounting for only 4%, underscores that the studied exotic pet market is largely dominated by species perceived as exotic from a European perspective.

### 3.2. Trading of Protected Species

**Figure 4:**
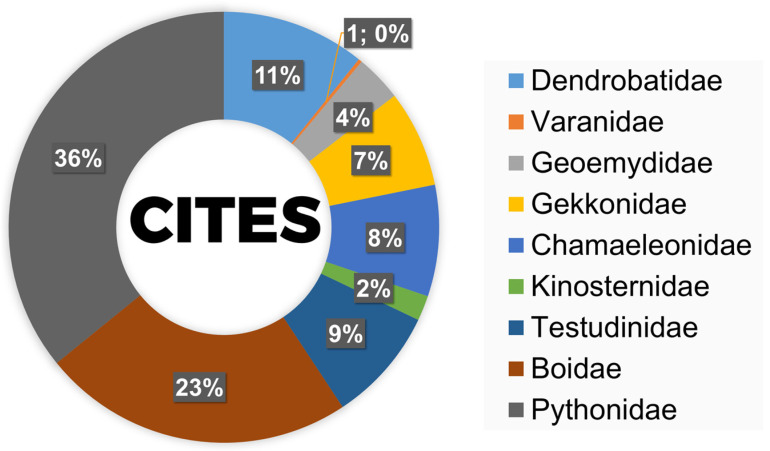
Taxonomic composition of CITES-listed species recorded in the studied exotic pet trade in Warsaw (Poland), expressed as the percentage share of families. Most species were listed in CITES Appendix II, while one species (*Mauremys sinensis*) was included in Appendix III. Detailed information on the assignment of individual species to CITES Appendices is provided in Supplementary Table S1.

A total of 50.59% of the species recorded during the fair were listed in the CITES Appendices (Figure 4). The vast majority of these were included in Appendix II, while only one species, the Chinese stripe-necked turtle (*Mauremys sinensis*), was listed in Appendix III. No species recorded in the study were included in Appendix I. None of the exhibitors’ booths had information on this subject. Although the event regulations did not require labeling species listed under CITES, they require exhibitors to have documents proving the legality of the animals.

An additional note was made as to whether the containers with the animals included information on their source, i.e., whether they were born in captivity (captive bred CB) or were imported (imported IM, wild caught WC). The largest part of the animals on offer was identified as CB (52.66% of all animals). Among all the species offered, only 1.98% were species marked as IM or WC on the boxes: *Varanus exanthematicus* (1 individual), *Phelsuma laticauda* (3 individuals), *Gonatodes albogularis* (3 individuals), *Trioceros jacksonii* (3 individuals), *Kinyongia boehmei* (1 individual), *Anolis biporcatus* (1 individual), and *Physignathus cocincinus* (4 individuals). The remaining 45.46% were animals on whose containers there was no information about their origin. However, the latter group includes popular species in breeding, e.g., *Boa imperator*, *Pantherophis guttatus*, *Heterodon nasicus*, *Python regius*, *Malayopython reticulatus*, *Python bivittatus*, *Mauremys sinensis*, and *Testudo horsfieldii*, which are being successfully bred in captivity. It is likely that these species originated from captive breeding but were not labeled as such. 

## 4. Discussion 

The results of this study demonstrate that reptiles, particularly squamates, dominated the exotic pet fair in Warsaw, which is consistent with broader patterns observed in the European and global exotic pet trade [5]. Reptiles are among the most frequently traded vertebrates in the international wildlife trade, and the diversity of reptile species present in the pet market has increased substantially over the past decades [1,3,17]. Analyses of global trade data indicate that thousands of reptile species are involved in the pet trade, with many of them traded legally through regulated markets such as captive breeding operations and commercial breeders [1]. In Europe, the reptile and amphibian pet trade has been shown to involve a particularly high diversity of species, including both widely bred species and less common taxa that occasionally appear in the market [10]. Studies of the European trade also suggest that collectors often seek visually distinctive or unusual species, which can drive demand for rare taxa and newly described species [10]. This pattern contributes to the increasing diversity of species appearing in the pet trade and may complicate the monitoring of wildlife trade and conservation status assessments.

Global analyses of the exotic pet trade further demonstrate that reptiles constitute a major component of the wildlife pet market, both in terms of number of species and volume of trade [8,10]. At the same time, the increasing diversity of traded species creates challenges for monitoring systems and conservation management, as many species enter the trade before their population status and conservation risks are fully understood [2].

An important aspect of the reptile trade is the growing role of captive breeding. Many species commonly encountered in the pet trade, such as the leopard gecko (*Eublepharis macularius*), ball python (*Python regius*), and corn snake (*Pantherophis guttatus*), are now widely bred in captivity and are considered relatively suitable for amateur keepers [6,14,18]. The high frequency of these species observed at the studied fair likely reflects their popularity, relatively well-established breeding techniques, and stable demand among hobbyists. Captive breeding may reduce pressure on wild populations when properly regulated and documented, although this depends on transparent and accurate labeling practices.

However, previous studies indicate that the origin of animals offered in the exotic pet trade is not always clearly communicated to buyers [4,6,19]. Inconsistent or incomplete labeling may obscure whether animals originate from captive breeding or wild collection and may limit the ability of consumers to make informed decisions. Similar concerns were identified in the present study, where nearly half of the animals lacked information regarding their origin. Improving the transparency of information provided at the point of sale could therefore contribute to better compliance with wildlife trade regulations and support responsible ownership of exotic species [6,20]. In this study the vast majority of animals were labeled with the common name, the Latin name, or both. Common names can be misleading due to their imprecision, although only popular species were labeled this way. Some containers were labeled with incorrect or outdated species names (for example *Python bivittatus* labeled as *Python molurus*), which could potentially mislead visitors, but these were isolated cases. Most labels also included information about sex and, where applicablel, the color morph. However, some labels contained information on age, source, husbandry, diet, habitat, or even adult size as well. Exhibitors could provide any missing information directly to visitors; however, expanding the information displayed on labels would undoubtedly make it easier for potential buyers to understand the characteristics and requirements of the species. Currently, based on the recommendations included in the codes of good practice of the Association of Polish Terrarium Keepers [19], the largest organizers of exotic animal fairs held in Poland require each container to be labeled with the Latin species name (and, where applicable, also the Polish common name), as well as information on the animal’s sex if it can be determined. At the time the fair took place, exhibitors were subject only to the internal regulations set by the individual organizer.

This study addresses a major knowledge gap in the understanding of the exotic pet trade in Poland. Previous research has focused primarily on invasive or potentially invasive taxa or on selected trade channels, such as the online trade in non-native amphibians [11,12,13]. As a result, the diversity of species present in the broader trade, particularly at exotic pet fairs, has remained largely undocumented. Similar data gaps exist in other European countries, and no previous studies have examined animals displayed at exotic pet fairs [10,21].

At the fair, five California kingsnakes (*Lampropeltis californiae*) were exhibited. This is a species to which, according to the European Union’s List of Invasive Alien Species of Union Concern [22], the regulations concerning *Lampropeltis getula* in a broader taxonomic sense apply (*L. getula*, among a few other species, refers on this list to *L. californiae*). However, at the time when the discussed fair took place, *L. getula* was not yet included on the list. The presence of species potentially associated with invasive taxa illustrates a broader issue related to the exotic pet trade, namely the risk of introduction of non-native species into natural ecosystems. Previous studies have shown that the pet trade is an important pathway for the introduction of alien reptiles and amphibians, either through accidental escapes or intentional releases by owners [4]. This problem has been documented in several regions, where species initially imported for the pet trade have subsequently established feral populations, creating ecological risks for native fauna and ecosystems [4].

Only a few species present at the fair were labeled with the provenance affix of the animal, such as Dendrobates auratus “Ancon Hill”. Unfortunately, it is common practice in the exotic pet trade to limit tagging to the species name alone. Labeling animals with exact geographic origin deserves greater attention due to the potential for hybridization of yet undescribed taxa or misidentified species. The importance of precise taxonomic and geographic information has also been highlighted in studies of the reptile and amphibian pet trade in Europe, which emphasize that incomplete labeling may contribute to taxonomic confusion and complicate monitoring of traded species [5]. Furthermore, analyses of global exotic pet markets indicate that increasing species diversity in trade can challenge existing monitoring systems and conservation assessments, particularly when information on animal origin is limited or absent [1,2,7,10].

Overall, the findings indicate that the exotic pet trade in Poland has the potential to operate in a relatively sustainable manner, largely based on captive breeding rather than import. Similar patterns have been reported in broader analyses of the global exotic pet trade, where captive breeding plays an increasingly important role in supplying commonly kept reptile species [10]. However, this potential is undermined by inconsistent labeling practices and insufficient communication of legal and conservation-related information. Strengthening supervision at exotic pet fairs, introducing standardized labeling requirements, and increasing public awareness could significantly improve transparency, animal welfare, and compliance with international conservation regulations. Such measures have been suggested as important tools for improving the governance and sustainability of the wildlife pet trade in Europe [10].

It should also be noted that the study was based on observations from a single exotic pet fair, and species composition may vary depending on the location, season, and specific event. Therefore, the results should be interpreted as a snapshot of the market rather than a comprehensive representation of the entire Polish exotic pet trade.

So far, no studies have investigated the diversity of species available within the Polish exotic pet trade. Consequently, the results obtained provide insight into the amphibian and reptile species kept in Poland, as well as the relative proportions of individual species within this hobby. This allows for an estimation of the popularity of the most commonly kept exotic vertebrate species in the country. Future studies should assess how information provided at the point of sale translates into actual husbandry practices and long-term health outcomes in captive reptiles and amphibians, as well as evaluate the effectiveness of labeling and regulatory measures in improving animal welfare and trade transparency. In addition, further research could investigate temporal variation in species composition at exotic pet fairs and other trade channels, as well as the role of online markets in shaping the availability of amphibians and reptiles in the European pet trade [2,7,10].

## 5. Conclusions

This study provides the first documented overview of reptile and amphibian species offered at an exotic pet fair in Poland, filling an important knowledge gap regarding the national exotic pet trade. The market was strongly dominated by reptiles, particularly squamates, with a small number of popular species accounting for a large proportion of individuals. Amphibians represented a smaller but taxonomically diverse component of the trade.

Although over half of the animals were labeled as captive-bred, nearly half lacked any information on origin, limiting the ability to assess the true scale of reliance on captive breeding versus wild sourcing. A substantial proportion of the species observed were listed in CITES Appendix II, yet no visible information on their legal or conservation status was provided at the point of sale. This indicates a significant gap in transparency and consumer awareness.

Inconsistent labeling practices concerning origin, legal status, and taxonomy may hinder informed purchasing decisions and reduce the effectiveness of conservation regulations. Standardized labeling requirements and improved oversight at exotic pet fairs would enhance transparency, support animal welfare, and strengthen compliance with international trade regulations. These findings highlight the need to integrate animal science principles into the regulation and monitoring of exotic pet markets.

Overall, the results suggest that while the trade may largely involve captive-bred individuals, insufficient documentation and communication of conservation-relevant information remain key challenges. The study establishes a baseline for future monitoring of species composition and management practices within the exotic pet trade and their implications for captive animal welfare in Poland and highlights the need for improved regulatory and educational measures.

## Data Availability

The data supporting the findings of this study are available in Supplementary Table S1.

## Supplementary Materials

The following supporting information can be downloaded at: https://www.mdpi.com/article/10.3390/ani16081138/s1, Table S1: Reptile and amphibian species offered for sale at an exotic pet fair in Warsaw (Poland), including origin and CITES status.
