# Supplementary material for: Composition and Legal Aspects of Reptiles and Amphibians Displayed at an Exotic Pet Fair in Warsaw (Poland)
Source: Animals (Basel). 2026 Apr 9;16(8):1138. doi: 10.3390/ani16081138 (PMC13113500; doi:10.3390/ani16081138)
Supplement: Supplementary file 1 [file animals-16-01138-s001.zip › animals-4164577-supplementary.pdf]

Table S1. Reptile and amphibian species offered for sale at an exotic pet fair in Warsaw (Poland), including origin and CITES status

Species name	Order	Suborder	Family	Number of individuals	Source (captive bred/ wild cought)	CITES statement	Additional information	Species native range
Phyllobates bicolor	Anura	Neobatrachia	Dendrobatidae	2	–	Appendix II CITES	–	western Colombia
***Dendrobates tinctorius* "citronella"**	Anura	Neobatrachia	Dendrobatidae	8	–	Appendix II CITES	–	Suriname
***Adelphobates galactonotus* "yellow"**	Anura	Neobatrachia	Dendrobatidae	1	–	Appendix II CITES	–	Brazil
Ranitomeya ventrimaculata	Anura	Neobatrachia	Dendrobatidae	2	–	Appendix II CITES	–	western Amazon Basin
***Dendrobates auratus* "Ancon Hill"**	Anura	Neobatrachia	Dendrobatidae	3	–	Appendix II CITES	–	Panama
***Dendrobates tinctorius* "robertus"**	Anura	Neobatrachia	Dendrobatidae	5	–	Appendix II CITES	–	southern Suriname
***Dendrobates tinctorius* "la fumeé"**	Anura	Neobatrachia	Dendrobatidae	2	–	Appendix II CITES	–	French Guiana
***Dendrobates tinctorius* "brazilian yellow head"**	Anura	Neobatrachia	Dendrobatidae	2	–	Appendix II CITES	–	Brazil
***Dendrobates tinctorius* "El Oro"**	Anura	Neobatrachia	Dendrobatidae	2	–	Appendix II CITES	–	southwest Ecuador
***Dendrobates auratus* "Costa Rica"**	Anura	Neobatrachia	Dendrobatidae	3	–	Appendix II CITES	–	Costa Rica
Dendrobates tinctorius	Anura	Neobatrachia	Dendrobatidae	5	–	Appendix II CITES	–	estern Guiana Shield

***Dendrobates tinctorius* "Kaw Mountain"**	Anura	Neobatrachia	Dendrobatidae	3	–	Appendix II CITES	–	French Guiana
***Dendrobates tinctorius* "azureus"**	Anura	Neobatrachia	Dendrobatidae	3	–	Appendix II CITES	–	Suriname
Sclerophrys mauritanica	Anura	Neobatrachia	Bufonidae	4	captive-bred	not listed	species description	North Africa
Rhinella marina	Anura	Neobatrachia	Bufonidae	2	–	not listed	species description	Central and northern South America
Incilius luetkenii	Anura	Neobatrachia	Bufonidae	1	captive-bred	not listed	species description	Central America
Duttaphrynus melanostictus	Anura	Neobatrachia	Bufonidae	2	–	not listed	species description	South and Southeast Asia
Ceratophrys cranwelli	Anura	Neobatrachia	Ceratophryidae	24	captive-bred	not listed	species description	Central South America
Trachycephalus resinifictrix	Anura	Neobatrachia	Hylidae	10	captive-bred	not listed	species description	Amazon Basin
Litoria caerulea	Anura	Neobatrachia	Pelodryadidae	11	captive-bred	not listed	species description	northern Australia and New Guinea
Hymenochirus boettgeri	Anura	Pipimorpha	Pipidae	17	–	not listed	–	Central Africa
Ambystoma tigrinum	Caudata	Salamandroidea	Ambystomatidae	2	–	not listed	species description	North America
Varanus exanthematicus	Squamata	Anguimorpha	Varanidae	1	wild caught	Appendix II CITES	–	West and Central Africa
Takydromus dorsalis	Squamata	Autarchoglossa	Lacertidae	4	captive-bred	not listed	species description	southern Japan
Phelsuma grandis	Squamata	Gekkota	Gekkonidae	13	captive-bred	Appendix II CITES	–	Madagascar
Uroplatus henkeli	Squamata	Gekkota	Gekkonidae	2	captive-bred	Appendix II CITES	–	Madagascar
Phelsuma klemmeri	Squamata	Gekkota	Gekkonidae	8	captive-bred	Appendix II CITES	–	northwestern Madagascar

Phelsuma laticauda	Squamata	Gekkota	Gekkonidae	3	captive-bred	Appendix II CITES	–	northern Madagascar
Phelsuma lineata	Squamata	Gekkota	Gekkonidae	1	wild caught	Appendix II CITES	–	Madagascar
Eublepharis macularius	Squamata	Gekkota	Eublepharidae	152	–	not listed	–	western South Asia
Correlophus auriculatus	Squamata	Gekkota	Diplodactylidae	7	–	not listed	–	New Caledonia
Correlophus sarasinorum	Squamata	Gekkota	Diplodactylidae	1	–	not listed	–	New Caledonia
Eurydactylodes vieillardi	Squamata	Gekkota	Diplodactylidae	3	–	not listed	–	New Caledonia
Correlophus ciliatus	Squamata	Gekkota	Diplodactylidae	68	captive-bred	not listed	–	New Caledonia
Lepidodactylus lugubris	Squamata	Gekkota	Gekkonidae	6	captive-bred	not listed	–	Indo-Pacific region
Gonatodes albogularis	Squamata	Gekkota	Sphaerodactylidae	3	wild caught	not listed	–	Central and northern South America
Nephrurus levis	Squamata	Gekkota	Carphodactylidae	1	–	not listed	–	Australia
Chamaeleo calyptratus	Squamata	Iguania	Chamaeleonidae	18	captive-bred	Appendix II CITES	–	southern Arabian Peninsula
Trioceros jacksonii	Squamata	Iguania	Chamaeleonidae	5	2 captive-bred, 3 wild caught	Appendix II CITES	–	East Africa
Furcifer pardalis	Squamata	Iguania	Chamaeleonidae	7	captive-bred	Appendix II CITES	–	Madagascar
Kinyongia boehmei	Squamata	Iguania	Chamaeleonidae	1	wild caught	Appendix II CITES	–	southeastern Kenya
Pogona vitticeps	Squamata	Iguania	Agamidae	25	captive-bred	not listed	–	central Australia

Pogona henrylawsoni	Squamata	Iguania	Agamidae	2	—	not listed	—	northeastern Australia
Physignathus cocincinus	Squamata	Iguania	Agamidae	4	captive-bred	not listed	—	Southeast Asia
Basiliscus plumifrons	Squamata	Iguania	Corytophanidae	1	—	not listed	—	Central America
Corytophanes hernandezii	Squamata	Iguania	Corytophanidae	1	—	not listed	—	Central America
Chamaeleolis porcus	Squamata	Iguania	Dactyloidae	1	captive-bred	not listed	—	Cuba
Anolis biporcatus	Squamata	Iguania	Dactyloidae	1	wild caught	not listed	—	Central and northern South America
Lacerta erhardii	Squamata	Lacertoidea	Lacertidae	2	captive-bred	not listed	—	southern and central Balkan Peninsula
Podarcis tauricus	Squamata	Lacertoidea	Lacertidae	2	captive-bred	not listed	—	southeastern Europe
Mochlus fernandi	Squamata	Scincoidea	Scincidae	9	5 captive-bred, 4 no data	not listed	—	West and Central Africa
Gerrhosaurus nigrolineatus	Squamata	Scincoidea	Gerrhosauridae	3	—	not listed	—	West and Central Africa
Boa imperator	Squamata	Serpentes	Boidae	49	—	Appendix II CITES	—	Central America
Boa imperator "Costa Rica"	Squamata	Serpentes	Boidae	11	—	Appendix II CITES	—	Costa Rica
Corallus hortulanus	Squamata	Serpentes	Boidae	8	—	Appendix II CITES	—	South America
Epicrates cenchria	Squamata	Serpentes	Boidae	12	—	Appendix II CITES	—	Central and South America
Epicrates cenchria × Epicrates maurus	Squamata	Serpentes	Boidae	2	—	Appendix II CITES	—	-
Eryx colubrinus	Squamata	Serpentes	Boidae	5	—	Appendix II CITES	—	North and East Africa

Python regius	Squamata	Serpentes	Pythonidae	113	–	Appendix II CITES	–	West and Central Africa
Malayopython reticulatus	Squamata	Serpentes	Pythonidae	12	–	Appendix II CITES	–	Southeast Asia
Python bivittatus	Squamata	Serpentes	Pythonidae	6	–	Appendix II CITES	–	Southeast Asia
Morelia spilota	Squamata	Serpentes	Pythonidae	1	–	Appendix II CITES	–	Australia and New Guinea
Morelia spilota cheynei	Squamata	Serpentes	Pythonidae	1	–	Appendix II CITES	–	eastern Australia
Pantherophis guttatus	Squamata	Serpentes	Colubridae	33	–	not listed	–	southeastern North America
Heterodon nasicus	Squamata	Serpentes	Colubridae	8	–	not listed	–	North America
Lampropeltis californiae	Squamata	Serpentes	Colubridae	5	–	not listed	–	western United States, northern Mexico
Lampropeltis triangulum nelsoni	Squamata	Serpentes	Colubridae	1	–	not listed	–	Mexico
Lampropeltis triangulum campbelli	Squamata	Serpentes	Colubridae	2	–	not listed	–	Mexico
Ahaetulla prasina	Squamata	Serpentes	Colubridae	2	–	not listed	species description	Australasia and the Indomalayan realm
Lycodon capucinus	Squamata	Serpentes	Colubridae	1	–	not listed	species description	Australasia and the Indomalayan realm
Xenochrophis vittatus	Squamata	Serpentes	Colubridae	1	–	not listed	species description	Indomalayan realm
Boaedon fuliginosus	Squamata	Serpentes	Lamprophiidae	4	–	not listed	–	sub-Saharan Africa
Pareas carinatus	Squamata	Serpentes	Pareidae	2	–	not listed	species description	Australasia and the Indomalayan realm
Macrochelys temminckii	Testudinata	Cryptodira	Chelydridae	1	–	Appendix II CITES	–	southeastern United States

Mauremys sinensis	Testudinata	Cryptodira	Geoemydidae	13	–	Appendix III CITES	species description	East Asia
Sternotherus odoratus	Testudinata	Cryptodira	Kinosternidae	7	–	Appendix II CITES	–	eastern North America
Centrochelys sulcata	Testudinata	Cryptodira	Testudinidae	8	4 captive-bred, 4 no data	Appendix II CITES	4 short species description, 4 Information on reaching large adult size (up to 50 kg)	southern Sahara and Sahel region (Africa)
Testudo hermanni	Testudinata	Cryptodira	Testudinidae	4	captive-bred	Appendix II CITES	–	southern Europe
Testudo horsfieldii	Testudinata	Cryptodira	Testudinidae	10	–	Appendix II CITES	–	Central Asia
Stigmochelys pardalis	Testudinata	Cryptodira	Testudinidae	4	–	Appendix II CITES	–	eastern and southern Africa
Kinixys belliana	Testudinata	Cryptodira	Testudinidae	6	–	Appendix II CITES	–	eastern and southern Africa
Pelodiscus sinensis	Testudinata	Cryptodira	Trionychidae	3	–	not listed	–	East Asia
Pelomedusa subrufa	Testudinata	Pleurodira	Pelomedusidae	4	–	not listed	species description	southern and central Africa
Pelusios castaneus	Testudinata	Pleurodira	Pelomedusidae	2	–	not listed	–	West Africa
